# Primary cilia and actin regulatory pathways in renal ciliopathies

**DOI:** 10.3389/fneph.2023.1331847

**Published:** 2024-01-16

**Authors:** Rita Kalot, Zachary Sentell, Thomas M. Kitzler, Elena Torban

**Affiliations:** ^1^ Department of Medicine and Department of Physiology, McGill University, Montreal, QC, Canada; ^2^ The Research Institute of the McGill University Health Center, Montreal, QC, Canada; ^3^ Department of Human Genetics, McGill University, Montreal, QC, Canada; ^4^ McGill University Health Center, Montreal, QC, Canada

**Keywords:** renal ciliopathies, genetics, primary cilium, ciliogenesis, actin regulation

## Abstract

Ciliopathies are a group of rare genetic disorders caused by defects to the structure or function of the primary cilium. They often affect multiple organs, leading to brain malformations, congenital heart defects, and anomalies of the retina or skeletal system. Kidney abnormalities are among the most frequent ciliopathic phenotypes manifesting as smaller, dysplastic, and cystic kidneys that are often accompanied by renal fibrosis. Many renal ciliopathies cause chronic kidney disease and often progress to end-stage renal disease, necessitating replacing therapies. There are more than 35 known ciliopathies; each is a rare hereditary condition, yet collectively they account for a significant proportion of chronic kidney disease worldwide. The primary cilium is a tiny microtubule-based organelle at the apex of almost all vertebrate cells. It serves as a “cellular antenna” surveying environment outside the cell and transducing this information inside the cell to trigger multiple signaling responses crucial for tissue morphogenesis and homeostasis. Hundreds of proteins and unique cellular mechanisms are involved in cilia formation. Recent evidence suggests that actin remodeling and regulation at the base of the primary cilium strongly impacts ciliogenesis. In this review, we provide an overview of the structure and function of the primary cilium, focusing on the role of actin cytoskeleton and its regulators in ciliogenesis. We then describe the key clinical, genetic, and molecular aspects of renal ciliopathies. We highlight what is known about actin regulation in the pathogenesis of these diseases with the aim to consider these recent molecular findings as potential therapeutic targets for renal ciliopathies.

## Introduction

Ciliopathies are a heterogeneous group of rare genetic disorders that arise due to defects in the structure or function of the primary cilium. The severity of the disease presentation in ciliopathies is highly variable and depends on the nature of the ciliary dysfunction, which, in turn, is dependent on both the affected gene and the type of genetic variant (i.e., mutation) involved. Disease-causing variants in over 180 human genes give rise to more than 35 established human ciliopathies, including Bardet Biedl Syndrome (BBS), Nephronophthisis (NPHP), Polycystic Kidney Disease (PKD), Retinitis Pigmentosa (RP), Joubert Syndrome (JBTS), Orofacial-Digital Syndrome (OFD) and many others. Ciliopathies often affect multiple organs, resulting in a range of congenital defects such as cardiac malformation, retinal degeneration, or skeletal defects. Kidney abnormalities are among the most frequent ciliopathic phenotypes, including renal hypo-dysplasia, fluid-filled cysts, or renal fibrosis (1–4). Most renal ciliopathies cause chronic kidney disease and progress to end-stage renal disease (ESRD). Although each disease is an individually rare hereditary condition, collectively, ciliopathies contribute significantly to chronic kidney disease worldwide with an associated burden on families, society and the health system.

The primary cilium is a specialized microtubule-based organelle that extends from the surface of almost all vertebrate cells. Although primary cilium was observed in mammalian cells as early as 1898 (5), for almost a hundred years after its discovery, it was thought to be a vestigial organelle that lacked functional properties (6). The role of the primary cilium as a key cellular signaling hub crucial for tissue morphogenesis and homeostasis was only discovered in the 1990s (7, 8).

The primary cilium is nucleated at the basal body which anchors it to the apical plasma membrane. The ciliary microtubule core, known as the axoneme, is covered by a ciliary membrane, which contains thousands of signaling molecules, receptors, channels, and transporters. Therefore, the primary cilium acts as a “cellular antenna” that protrudes outside of the cell, senses the surrounding extracellular environment, and transduces the information inside the cell to trigger signaling pathways that govern important cellular responses (9). The primary cilium integrates different cellular components such as cytoskeletal actin and microtubule networks with unique cellular trafficking machinery and a repertoire of signaling molecules. Disruptions to these functions cause human ciliopathies including diseases with kidney involvement (10).

In this review, we will provide an overview of the structure and function of the primary cilium and describe the clinical, genetic and molecular aspects of the renal ciliopathies. We will emphasize the role of the actin cytoskeleton at the primary cilium and describe recent molecular findings that might be of therapeutic importance for renal ciliopathies, as summarized in **Box 1**.

## Primary cilia and ciliogenesis

### Motile and sensory cilia

Cilia have evolved to accomplish two major functions, motility and sensation, and, accordingly, are classified as motile and sensory (11). Motile cilia (or flagella) were the first organelles ever observed in mid-16^th^ century, when Antony Van Leeuwenhoek used the simple light microscope he invented to visualize a tiny protozoan organism in rainwater that rapidly moved with the help of multiple miniature “legs” (12). These “legs” were later named “cilia” or “eyelashes” in Latin for the plural or “cilium” or an “eyelash” for a single structure (12). Motile cilia are found in a subset of tissues such as the bronchi of the lungs, cells lining brain ventricles and other organs (13), and are characterized by their ability to move in a coordinated manner to propel fluid or cells in the extracellular space (14). The axoneme of the motile cilium is composed of 9 microtubular duplets, which are the extensions of the 9 microtubular triplets that make up the basal body. In addition, motile cilia have an inner central pair of microtubules, an arrangement described as (9 + 2) and other proteins that enable motile cilia to beat coherently and directionally (15). Unlike motile cilia, a sensory, or primary, cilium is solitary (present in a single copy per cell), extending from the apical surface of almost all cells, including kidney or lung epithelia. The primary cilium lacks the central microtubule pair and the molecules associated with the ciliary motility. The primary cilium adopts a (9 + 0) arrangement and harbors an abundance of signaling molecules within its membrane enabling it to function as a signaling hub (16–18). Motile and primary cilia share a plethora of common structural proteins, however, many proteins, especially those required for ciliary motility or sensory function, are unique to each type (18).

### The early stage of ciliogenesis

Cilia biogenesis, referred to as “ciliogenesis” hereafter, begins when a cell exits the cell cycle. Upon cell cycle exit in G0, the mother/daughter centriole pair, that serves as the microtubule organizing center (MTOC) during cell division, is liberated and the mother centriole undergoes maturation by acquiring morphologically and functionally distinct distal and subdistal appendages; it then ascends towards and docks beneath the apical surface, thereby becoming the basal body (19). The distal appendages are recruited to the basal body by centrosomal proteins and are required for anchoring the basal body to the apical cell membrane, while subdistal appendages help nucleate microtubules to build ciliary axoneme (20–23). Mother centriole maturation is governed by multiple proteins, many of which are linked to human ciliopathies. For example, mutations in two of the genes encoding distal appendage proteins, CEP164 and CEP83, cause nephronophthisis phenotypes, while the distortion of centrosomal proteins OFD1 and C2CD3 (both are also engaged in the distal appendage assembly) is linked to JBTS, OFD, and RP (9). Additionally, centriolar satellites, which are electron-dense structures that move around the basal body, maintain the centrosome and cilium by facilitating the transport of ciliary and centrosomal proteins (9).

### Pathways of ciliogenesis

**Figure 1:**
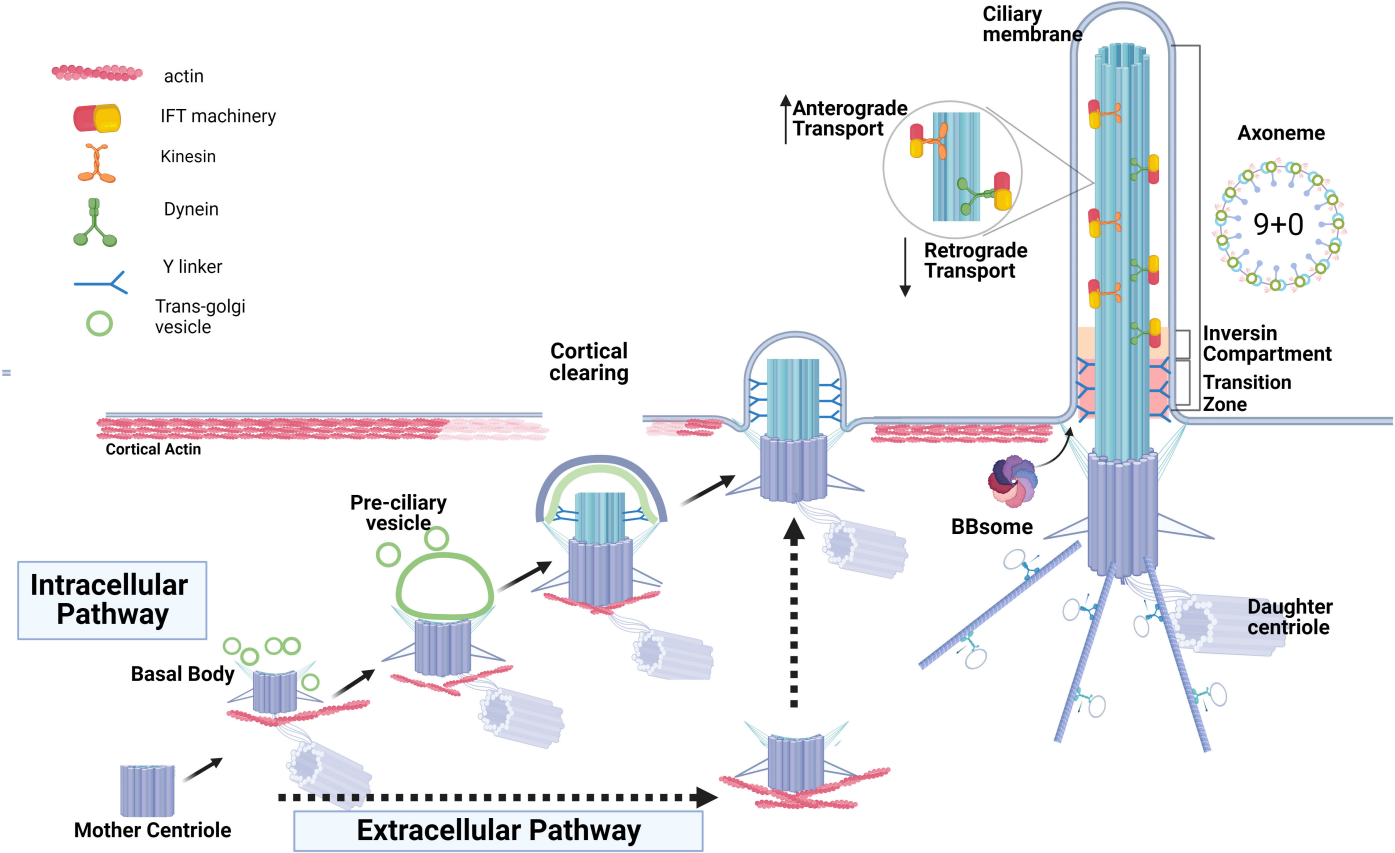
Two routes of ciliogenesis and the ciliary structures. The formation of the primary cilium by *intracellular* pathway commences when the mother centriole matures by acquiring distal and sub-distal appendages and transforming into the basal body. This is followed by the formation and enlargement of a pre-ciliary vesicle around the mother centriole via fusion of centriole’s appendages with the ciliary-protein cargo vesicles originating from trans-Golgi. The basal body ascends and then docks to the apical membrane and the ciliary vesicle fuses with the plasma membrane to form the ciliary membrane and the ciliary pocket. The *extracellular* route of ciliogenesis in the epithelial cells (intermittent black arrows) is characterized by direct docking of the basal body to the apical membrane and cilia elongation without the formation of a ciliary pocket. Basal body docking is preceded by clearing of cortical actin at the cell apex, an obligatory step permitting ciliogenesis. Docked basal body templates axoneme extension, and cilia growth via bi-directional anterograde and retrograde Intraflagella transport, IFT, is depicted. The inversin compartment and the transition zone immediately above the basal body form a diffusion barrier to control protein content within the ciliary shaft and ciliary membrane.

Depending on the cell type, two pathways of ciliogenesis, “intracellular” and “extracellular”, have been described (24, 25) (**Figure 1**). The intracellular pathway mostly occurs in non-polarized cells, although it was described in some epithelial cells as well (26). It begins with the formation of a large ciliary vesicle which encloses the mother centriole. The ciliary vesicle forms via trafficking of Golgi-derived cargo protein vesicles toward the distal appendages of the mother centriole and fusing with them. The ciliary vesicle grows and then fuses with the plasma membrane forming the ciliary membrane and the ciliary pocket. The latter is a membrane invagination that keeps the primary cilium submerged in the cytoplasm and is a site for ciliary protein trafficking and active endocytosis (24, 27, 28). On the other hand, polarized epithelial cells (such as kidney epithelium) utilize the extracellular pathway to form the primary cilium at the cellular apex. In this pathway, the primary cilium is formed only after the mother centriole has acquired its distal and subdistal appendages and docked directly to the plasma membrane. The core ciliogenic machinery is shared between the intracellular and extracellular pathways, however, some differences between the two routes exist (reviewed in (28)). For example, the exocyst complex is critical for ciliogenesis in the polarized kidney epithelial cells but is dispensable in the retinal pigmental epithelial RPE-1 cells employing intracellular ciliogenesis (29). Emerging evidence suggests that defects attributed to each specific ciliogenic pathway might contribute to different ciliopathy phenotypes or tissue-specific defects (28, 30, 31).

Box 1 Key points.

- The primary cilium is a specialized microtubule-based organelle that extends from the surface of almost all vertebrate cells and functions as a key signaling nexus for tissue morphogenesis and homeostasis.
- Defects to primary cilium formation and function cause a heterogeneous group of hereditary disorders known as ciliopathies. Ciliopathies that feature kidney abnormalities (renal ciliopathies) are among the most common.
- Recent evidence has revealed complex actin dynamics at the basal body that critically affect primary cilium formation.
- Many ciliopathy-associated proteins directly or indirectly regulate actin-cytoskeletal dynamics during cilium formation.
- Actin dynamics are disrupted across renal ciliopathy disorders, contributing to cystic transformation, fibrosis, and renal malformations due to dysregulated cilium-dependent developmental signaling processes.
- The actin-dependent mechanisms in renal ciliopathies are an emerging field that promises to uncover important mechanistic aspects of disease pathogenesis and discover new potential therapeutic targets.

### Initiation of ciliogenesis

To initiate ciliogenesis, the removal of the CP110-CEP97 protein complex from the mother centriole is required (32). This complex is localized at the distal end of centrioles and exerts control over centriole length by inhibiting centriolar microtubule extension (33–35). The elimination of CP110 is important for the transformation of the mother centriole into the basal body, as retention of CP110 at the mother centriole blocks cilium formation (36–38). Membrane remodeling occurs directly above the site of future basal body docking and is known as “cortical clearing”; it is an obligatory step that precedes docking in both intracellular and extracellular ciliogenesis pathways and is followed by basal body microtubule extension and assembly of the ciliary axoneme (39).

## Primary cilia compartments

### Transition zone

The transition zone (TZ) is a highly organized compartment at the base of the cilium, acting as a gate that controls protein entry to and exit from the ciliary compartment (40, 41). The TZ consists of transition fibers connecting the distal part of the basal body to the base of the ciliary membrane and Y-shaped structures (known as Y-links) (**Figure 1**). The Y-links serve as physical connectors between the microtubular doublet of axoneme and the ciliary membrane (40, 42). The precise mechanisms underlying the formation of the TZ and how it effectively carries out its regulatory functions are not well understood and remain areas of ongoing active investigation (43). Transition fibers act as docking sites for ciliary trafficking machinery (44, 45). In addition, disrupting the formation of transition fibers impedes basal body docking and blocks ciliogenesis at an early stage (21, 46). The TZ is essential to the function and integrity of the primary cilium, notably to regulate the downstream cellular pathways such as Sonic Hedgehog (SHH) and WNT signaling pathways (47).

### Components of the transition zone

The transition zone is composed of a complex network of several protein compartments with at least 28 proteins associated with the TZ (9) (**Figure 1**). NPHP1, NPHP4 and NPHP8 form the nephrocystin module (NPHP1-4-8) involved in apical organization of renal epithelial cells (48). The nephrocystin module interacts with the CEP290 module (CEP290-NPHP5) at the basal body, as well as with the MKS module of the transition zone (43). The MKS module is critical to ciliogenesis and SHH signaling and is composed of several evolutionarily conserved proteins that are associated with severe ciliopathy phenotypes. INVS, NPHP3, NEK8, and ANKS6 localize to the inversin compartment, which forms distally to the transition zone along the axoneme (49). Disruption of any of these modules leads to the abnormal formation and function of the primary cilium. For example, the absence of CEP290 results in TZ malfunction that leads to alterations in cilia composition. Additionally, an increasing number of proteins are being recognized as reliant on an intact TZ for their precise localization within the cilium (47–50). Proximity ligation assays broadened and refined the protein-protein interaction network at TZ, confirming the majority of well-known TZ proteins and revealing novel prospective TZ protein candidates and potential linkages to other ciliary proteins excluded from the TZ (51–54).

### Intraflagellar transport

The cilium is built via a unique, evolutionary-conserved cellular machinery known as intraflagellar transport or IFT, that is dedicated to the transport of ciliary proteins and lipid components toward and within the primary cilium (30, 55). IFT machinery is composed of anterograde transport complexes (IFT-B) powered by kinesin motors (56, 57) and retrograde transport complexes (IFT-A), which are mainly dynein motor-dependent (58, 59). Six proteins constitute the IFT-A group, while sixteen proteins were identified in the IFT-B group. Proteins of both IFT-B and IFT-A complexes are enriched with domains that enable extensive interactions with multiple partners, as observed in IFT-IFT and IFT-cargo interactions (60). Utilizing total internal reflection fluorescence microscopy and photobleaching techniques, it was shown that a solitary anterograde train pauses upon reaching the ciliary tip and subsequently divides into multiple retrograde trains (61). This observation implies a significant structural reconfiguration of the trains and a dynamic cross-talk between the IFT-A and IFT-B proteins, although the details of how the switch from anterograde to retrograde transport occurs are mainly unknown (62). Genetic variants in several IFT machinery components are associated with multi-systemic ciliopathies including BBS (63), JBTS (64), OFD1 (65), and NPHP (66) signifying the importance of this machinery in maintaining the cilium.

### The Bardet-Biedl syndrome protein complex

The BBSome is a ciliary trafficking protein complex composed of eight BBS protein subunits (BBS1, 2, 4, 5, 7, 8, 9 and BBIP10) that has similarity to the coat protein trafficking systems (67) (68). Several additional proteins act as chaperonins and are required for BBSome assembly (69). The BBSome constituents are localized in the cilium *proper*, the basal body, and centriolar satellites. They regulate both ciliary cargo protein transport and signaling processes. The BBSome’s role is pivotal for the ciliary entry of many signaling molecules: e.g. SMO protein (a key part of the SHH signaling pathway) or several G-protein-coupled receptors which are absent from the cilium when the BBSome is malfunctional (70, 71). Likewise, somatostatin receptor type 3 is absent from the cilia of hippocampal neurons in *Bbs2*- or *Bbs4*-mutant mice (71). In addition, the BBSome maintains microtubule stability by preventing histone deacetylase HDAC6-mediated microtubule deacetylation and destabilization through its BBIP10 subunit (which binds to and sequesters the HDAC6) (68). More recent findings also highlight the BBSome’s involvement in ciliary protein exit thus regulating the equilibrium of ciliary receptor concentration and their localization on ciliary membrane (72, 73). Mutations in the genes encoding BBSome subunits and BBSome chaperonins cause pleiotropic Bardet-Biedl syndrome (72, 74, 75) (discussed below). Overall, proper cilia formation depends on the coordinated interplay between the different ciliary compartments and their prospective components.

## The roles of actin in ciliogenesis

### Hints of actin involvement in the formation of primary cilia

**Figure 2:**
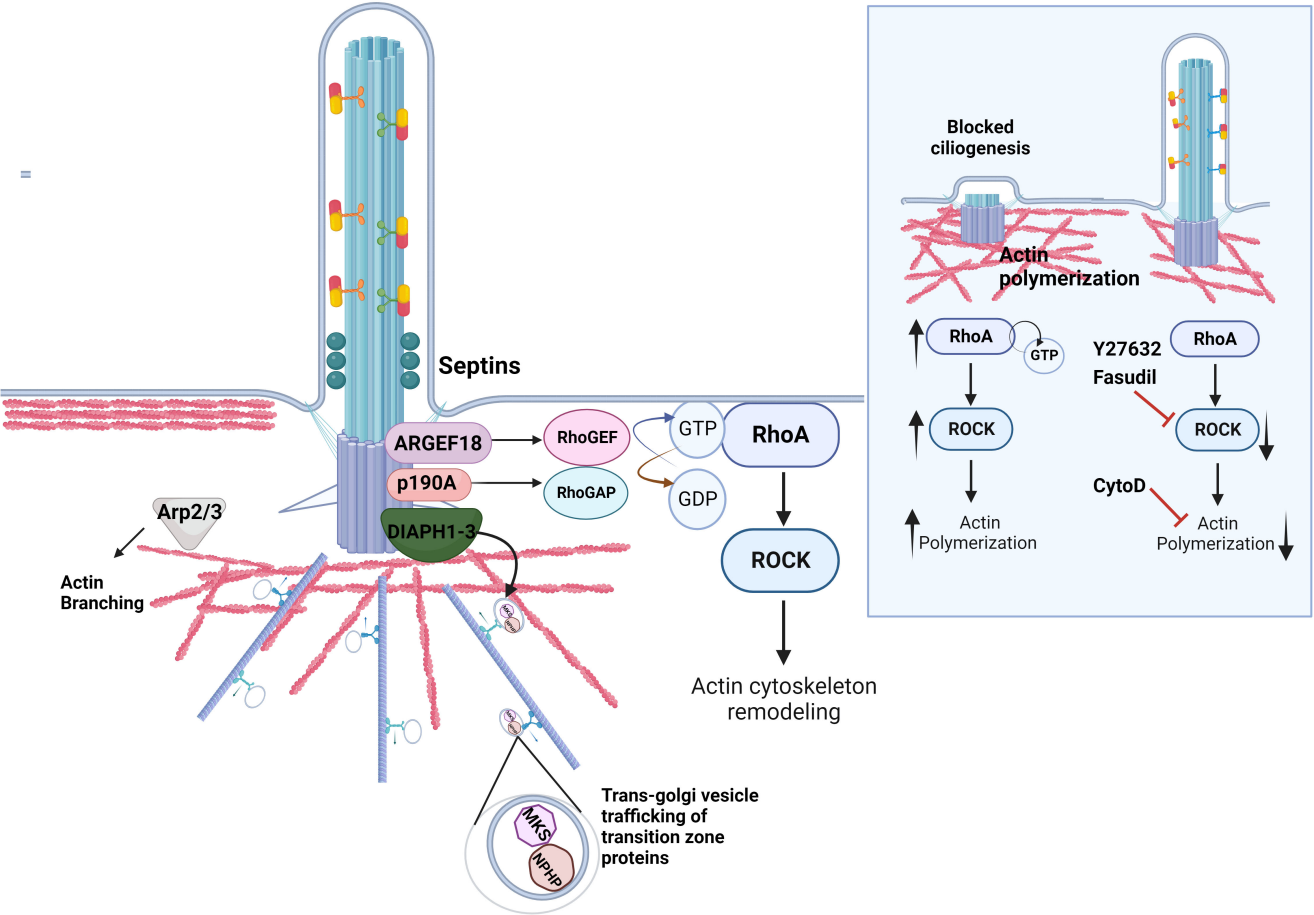
Regulation of actin dynamics in ciliogenesis. The actin cytoskeleton at the ciliary base contributes to cilia assembly and function. A set of actin regulatory proteins including DIAPH1-3 family of formins is important for the proper transport of ciliary proteins as well as actin nucleation and polymerization. The proteins of the family of Rho GTPases RhoA directly impact ciliogenesis by regulating actin dynamics at the ciliary base. RhoA cycles between an active GTP-bound and an inactive GDP-bound form with the help of RhoGEFs and RhoGAPs, respectively. Septins localize to the transition zone of the primary cilium, contribute to the diffusion barrier formation and influence the activity of the RhoA and its effector RhoA kinase, ROCK, at the basal body via ARHGEF18. p190A RhoGAP localizes to the basal body and inhibits excessive RhoA activation. Similarly, Arp2/3 complex mediates F-actin branching, which inhibits ciliogenesis. *Inlet:* Excessive RhoA activity due to mutations in the genes that regulate the Rho pathway lead to increased actin polymerization around the ciliary base, which hinders the transport of ciliary protein cargo and retains disassembly factors, inhibiting ciliation. The rescue of ciliogenesis can be achieved by utilizing the ROCK inhibitors Fasudil and Y2732, or the actin depolymerizing agent Cytochalasin D.

In the last decade, a particular role for the actin cytoskeleton in ciliogenesis emerged (10). For example, vesicle trafficking, basal body docking, centrosome positioning, and intraflagellar transport are all actin-dependent processes. The primary cilium is also rich in actin regulatory proteins, and genetic or pharmaceutical disruption of actin dynamics may negatively or positively affect ciliation in cells *in vitro *[reviewed in (10)] (**Figure 2**). Actin localization within the photoreceptor connecting cilium was initially observed by Chaitin et al. as early as 1984 (76). Mammalian photoreceptors consist of an inner segment, the cell body where proteins are synthesized, and the outer segment, where photo-sensing takes place. These segments are connected by the “connecting” cilium that uses IFT machinery to move proteins from the inner to the outer segment. Liu et al. demonstrated that actin bundles act as tracks, facilitating the transportation of the periciliary membrane complex to the basal body located at the base of the connecting cilium (77, 78). This study for the first time revealed a potentially important role of actin cytoskeleton in the regulation of primary cilium dynamics.

### Genomic and proteomic screens defining the ciliome

To understand the regulatory mechanisms governing the formation of the primary cilium, several ciliogenesis modulator screens were carried out to identify key drivers and repressors influencing the formation and maintenance of primary cilia (79, 80) and to demarcate the ciliome, which represents the extensive repertoire of ciliary proteins currently counting over 1000 molecules (81). Independent studies such as Syscilia Gold standard (82), CiliaCarta (83), and Cildb (9, 84) were dedicated to defining the ciliome and identifying candidate ciliary genes using genomic, proteomic, transcriptomic, and evolutionary data, often integrating Bayesian statistics for predictive scoring of ciliary function. Moreover, recent research efforts provided valuable transcriptomic resources for curated primary ciliomes, establishing tissue and temporal specificity within various subgroups of differentially expressed genes associated with ciliary structures (85).

The involvement of actin in ciliogenesis was revealed in a comprehensive functional high-throughput RNA interference screen aiming to define genes involved in ciliogenesis (79). The screen evaluated a total of 7,784 therapeutically relevant human genes and revealed several molecules related to actin dynamics and vesicle trafficking required for ciliogenesis. Among these, gelsolin, an actin filament severing protein, emerged as a positive regulator, while the actin nucleating proteins of the Arp2/3 complex, that drive actin branching, were identified as negative regulators. Additionally, cilia-associated proteomics, combined with proximity-based biotinylation (Bio-ID) of the cilium-targeted APEX2 peroxidase enzyme, enabled Kohli et al. to detect a high stoichiometry of actin-binding proteins (86). Notably, actinin-1 & 4, tropomyosins-3 & 4, ezrin, gelsolin, cortactin and other actin regulatory proteins were found to be abundantly associated with cilia. Gupta et al. employed a BioID approach to systematically map the centrosome-cilium interface (53). This study generated a protein topology network of over 7,000 interactions with 58 bait proteins. The analysis of interactions and phenotypic profiles revealed protein modules involved in centriole duplication ciliogenesis and centriolar satellite biogenesis. Importantly, the study highlighted extensive novel interactions between centrosome-cilium interface proteins and actin regulators such as RhoA, ARHGAP21, and DIAPH3 (53). Additionally, Choksi et al. (87) undertook genome-wide expression profiling and large-scale functional studies in a zebrafish model to describe a comprehensive network of motile cilia genes controlled by Foxj1, the master regulator of cilia biogenesis. Their gene ontology analysis revealed a set of 78 cytoskeletal proteins, including the actin regulator ARHGEF18. Ultrastructural studies found actin within cilia *prop*er where it participates in regulating ciliary length by “decapitating” the ciliary tip (87). In fact, localized actin is essential for ectocytosis (vesicle shedding), aiding G protein-coupled receptor recycling at the ciliary tip (88). Overall, these studies uncovered an intriguing phenomenon: actin polymerization and branching must be precisely regulated spatially and timely to allow the formation of primary cilium, its function and maintenance.

## Actin-dependent mechanisms of ciliogenesis

### Actin dynamics at early stages of ciliogenesis

Actin dynamics are critical for ciliogenesis during the early stages since actin governs directional transportation of the mother centriole (future basal body) to the appropriate cell cortex (89). Subsequent steps include the anchoring of the basal body to the underlying actin cytoskeleton through focal adhesion proteins, followed by localized actin clearing at the apical surface and microtubule nucleation (39, 90). Prior to the basal body docking, a reduction in cortical actin was shown to positively affect ciliary vesicle trafficking and to promote axoneme extension (39). Actin remodeling at the cell’s apex is particularly important in polarized epithelial cells, in which the basal body docks directly to the apical membrane to initiate ciliogenesis. Although actin is asymmetrically distributed in all cells, the polarized epithelium is characterized by a thick cortical actin layer, which is cleared as the basal body docking occurs (39). Several molecules including RAB19 and lysosomal membrane-tethering HOPS complex are implicated in cortical clearing in the polarized dog kidney Madine-Darby collecting duct epithelial cells (extracellular pathway) as well as in the retinal epithelial cells RPE-1, which utilize intracellular ciliogenesis route (39, 91). However, the precise sequence of events during cortical clearing or whether a specific complement of actin regulatory proteins exists in polarized vs non-polarized cells is still unknown. On the contrary, a thick actin network is required in multiciliated cells, in which thousands of basal bodies per cell find orderly integration within a dense cortical actin mesh, directly anchoring to the actin and microtubular cytoskeleton for proper positioning and organized planar alighment (13, 92).

During ciliogenesis, actin reorganization coincides with microtubule polymerization, stabilization, and modification (10). Actomyosin contraction and the asymmetry of the stable microtubule network within the cell generate the force required to push the centrosome to the apical surface. The crosstalk between actin and microtubule networks is tightly regulated by cytoskeleton-associated proteins, many of which play crucial roles in cilium formation and maintenance (10, 93) Furthermore, the transport of vesicle cargo powered by myosin II and myosin Va (actin regulatory protein) delivers essential ciliary membrane components including Arl13b (10), the proteins of exocyst complex, the BBSome and others to the base of the growing cilia. Myosin Va is involved at the earliest stages of ciliogenesis, where it transports preciliary vesicles through its association with Rab 8 and Rab11 small GTPases (94) that mark vesicle trafficking route to the basal body (10).

### Cilia disassembly

Actin at the basal body is also important for cilia disassembly (95). Cilia disassembly occurs when the cell re-enters the cell cycle or during injury and epithelia-to-mesenchyme transition, e.g., in fibrosis or cancer (95–97). Three mitosis-regulating kinase families, Aurora A kinase (AURKA), Polo kinase (PLK1) and NimA-related kinase (NEK) and their associated proteins have been implicated in this process (98–100). Both AURKA and PLK1 phosphorylate and activate histone deacetylase 6 (HDAC6), which deacetylates axonemal tubulin, thereby destabilizing the microtubule-based ciliary core and promoting cilia resorption (98, 99). NEK family members phosphorylate other proteins, e.g. Kif24, that also favors tubulin depolymerization (100). Actin polymerization, especially actin branching, appears to hasten cilia disassembly (95). Although the detailed mechanisms remain unclear, it is believed that the centrosomal polymerized actin plays a role in retaining disassembly determinants AURKA, PLK1 and NEK at the base of the cilium leading to an enhanced cilia disassembly (95). Interestingly, translocation of Hippo pathway downstream effectors YAP and TAZ to the nucleus is cued by the actin polymerization cascade and results in increased expression of AURKA and PLK1 accompanied by suppression of ciliogenesis (101). This event appears to reinforce a feedback loop where excessive RhoA and actin polymerization trigger mechanisms of cilia disassembly. On the contrary, cytoplasmic sequestration of YAP/TAZ from the nucleus increased ciliogenesis and lowered AURKA and PLK1 expression levels, indicating that YAP/TAZ transcriptionally controls the expression of some disassembly factors (101).

## Molecular determinants of actin regulation at the primary cilium

### DIAPH family of formins

Multiple actin regulatory proteins control actin dynamics throughout the different stages of ciliogenesis [reviewed in (28, 95)] (**Figure 2**). Actin filament formation requires actin nucleation factors such as formins. The DIAPH family of formins, which includes DIAPH1, DIAPH2 and DIAPH3, features N-terminal GTPase-binding domains that interact with and are activated by small GTP-binding proteins of the Rho subfamily. Polander et al. showed that depleting DIAPH1 by RNA interference led to impaired ciliogenesis and reduced ciliary length. Reciprocally, targeting DIAPH1 to the basal body using centrin or PACT domains induced elongation of cilia and the formation of bulbous ciliary tips, suggesting that DIAPH1 likely acts in regulating ciliary vesicle trafficking. Similarly, DIAPH2 and DIAPH3 localize at the ciliary base and are implicated in the regulation of cilia maintenance by mediating post-Golgi and recycling endosomal vesicle trafficking (102). Depletion of DIAPH2 and DIAPH3 leads to decreased cilia length and accumulation of the ciliary structural protein IFT20 and small GTPase Rab11 at the cilium. On the contrary, both IFT20 and Rab11 levels increase upon targeting DIAPH2 and DIAPH3 to the ciliary base (103).

### Rho family of GTPases and its regulators

The establishment of the actin network in ciliated cells critically relies on small GTPases that act as regulators of actin dynamics and are capable of orchestrating pathways for actin assembly and remodeling, microtubule organization, as well as contributing to activation of essential transcription factors that trigger cell differentiation (104, 105). The Rho family of GTPases counts more than 20 members (106); RhoA, Rac1, and Cdc42 are the most studied. Functioning as molecular switches, these GTPases cycle between their active, GTP-bound state, and their inactive, GDP-bound configuration. The cyclic transition depends on three distinct classes of molecules: 1) guanine nucleotide exchange factors (GEFs), responsible for catalyzing the exchange of GDP for GTP, thereby activating Rho-family proteins; 2) GTPase activating proteins (GAPs), which expedite the inherent GTP hydrolytic activity of the Rho-family, thereby tempering the signaling cascade and 3) Guanine nucleotide dissociation inhibitors (GDIs), that bind to Rho GTPases and stabilize them in their inactive states (106–108).

Using primary cultures of mouse multiciliated tracheal epithelial cells (mTEC), Pan et al. showed that RhoA plays a critical role in the development of a distinct apical actin network (109). The apical actin web is indispensable for the docking of basal bodies, their uniform tilting (planar polarization) and the subsequent elongation of the ciliary axoneme. Park et al. showed that the Rho GTPase activation by planar cell polarity proteins Dishevelled and Inturned is required for basal body docking to the apical plasma membrane of multiciliated *Xenopus* cells (110). It is plausible that the regulation of Rho activity for apical docking and planar polarization of basal bodies involves distinct RhoA regulatory proteins, a notion reinforced by observations that knockdown of ARHGEF11 triggers various embryonic anomalies indicative of impaired cilia-mediated fluid flow, likely due to deregulation of basal body planar alignment and, as a consequence, lack of uniform directional ciliary beating (110).

Employing an ENU mutagenesis strategy, Steward et al. generated a mouse model displaying renal hypoplasia associated with glomerular cysts, which stemmed from a point mutation in the *ArhGAP35* gene that encodes p190A RHOGAP, one of the main mammalian GAPs (111). This loss-of-function mutation induced mis-localization of p190ARHOGAP away from the basal body, ultimately yielding escalated RhoA activity, increased actin polymerization at the ciliary base and a curtailed ciliogenesis seen as both diminished percentage of ciliated cells and shorter primary cilia in mouse proximal tubules as well as mouse embryonic fibroblasts (MEFs). Importantly, pharmacological inhibition of RhoA kinase (ROCK) activity rescued ciliary length in mutant MEFs (111). Building on these findings, Streets et al. screened several ARHGAPs and identified ARHGAP5, -29, and -35 as required for cell ciliation and ciliary length (112). After analyzing *PKD1* homozygous cells it was found that p190A RHOGAP was absent at the basal body of these mutant cells, which also featured elevated RhoA activity at the basal body and shorter cilia. In conclusion, the lack of centrosomal PC1-ARHGAP35 interactions in the *PKD1* mutant cells might contribute to cyst formation in ADPKD (112).

### Branched filamentous actin

Branched F-actin, which is primarily distributed in the cell cortex, is nucleated by the ARP2/3 complex. Notably, silencing the key component of the ARP2/3 complex, ACTR3 (also known as ARP3) led to a marked increase in ciliary length and facilitated ciliogenesis in hTERT-RPE cells (79). Similarly, Coa et al. showed that overexpression of mir-129-3p not only stimulated ciliation in proliferating mammalian cells but the primary cilia were also elongated (38). These effects were caused by simultaneous downregulation of four positive regulators of branched F-actin: ABLIM1, ABLIM3, TOCA1, and ARP2. Furthermore, the modulation of Arp2/3 complex activity led to the accumulation of ciliary vesicles in the cilia *proper*, suggesting that Arp2/3 complex plays a role in IFT-B distribution within cilia, therefore highlighting its significance in regulating both IFT entry into the cilia and ciliary length (113).

### Septins

*S*eptins are the family of GTP-binding scaffold proteins that associate with the cell membrane and cytoskeleton (114). During ciliogenesis, septins participate in the formation of the diffusion barrier, partitioning the ciliary membrane from the rest of the apical plasma membrane. Septins also regulate RhoA activity and actin polymerization facilitating trafficking of ciliary cargo proteins (13). For example, SEPTIN9 enhances the activity of ARHGEF18, which in turn activates RhoA (115). In this context, activated RhoA is required for the assembly of the complete exocyst complex (116), which orchestrates transport of post-Golgi vesicles containing transition zone proteins of the NPHP and MKS complexes (115, 117).

### Inhibition of actin polymerization pathway and ciliogenesis

In 2010, Kim et al. reported that the inhibition of actin assembly through drug intervention facilitates ciliogenesis in immortalized human retinal pigmented epithelial cells (hTERT-RPE) (79). This effect was attributed to the stabilization of the pericentrosomal preciliary compartment (PPC), a vesiculotubular structure responsible for storing transmembrane proteins needed for the initial phases of ciliogenesis. Actin depolymerization halts vesicle transport, causing vesicles to accumulate within the PPC. Furthermore, actin depolymerization at the base of the cilium leads to the formation of actin nodes that exhibit a marked affinity for delivering ciliary membrane cargo proteins to the ciliary vesicle. Thus, the reduction of actin polymerization facilitates the efficient delivery of cargo to the ciliary vesicle (79). On the contrary, actin polymerization, and especially actin branching, establishes a physical barrier to vesicle transport and ciliary membrane remodeling, inhibiting ciliogenesis (95) (**Figure 2**). Thus, actin dynamics in ciliogenesis constitute a remarkable phenomenon where the depolymerization of actin serves to promote both the assembly and elongation of cilia. For instance, the application of Cytochalasin D (CytoD), a small molecule that induces the depolymerization of filamentous (F)-actin, rapidly triggers both the formation of primary cilia and an excessive elongation of cilia likely through enhancing trafficking of essential ciliary proteins such as Arl13b and the dynamics of IFT machinery (79, 118). Remarkably, CytoD induces ciliogenesis even under conditions that typically trigger ciliary disassembly, such as serum stimulation of cultured cells (79). Interestingly, CytoD seems to also cause cytoplasmic retention of YAP and TAZ, thereby indirectly inhibiting transcription of ciliary disassembly factors (101). Ciliary elongation was also achieved by treating cells with a selective RhoA kinase inhibitor Y27632 (119). On the contrary, factors that induce branched actin assembly exert inhibitory effects on ciliogenesis (38, 120, 121). However, suppression of branched-actin regulators such as Arp2 via microRNA-mediated approaches (38) or treatment with a specific Arp2/3 inhibitor enhances trafficking of ciliary proteins (113).

In summary, actin polymerization affects different aspects of cilia formation and maintenance. The intricate interplay between actin dynamics and ciliogenesis relies on multiple actin regulatory proteins that orchestrate the balance between elongation and disassembly of the cilium, ultimately ensuring fine-tuning of cilia formation, maintenance, and function.

## Renal ciliopathies: molecular mechanisms of disease and actin regulation

### Preamble

**Figure 3:**
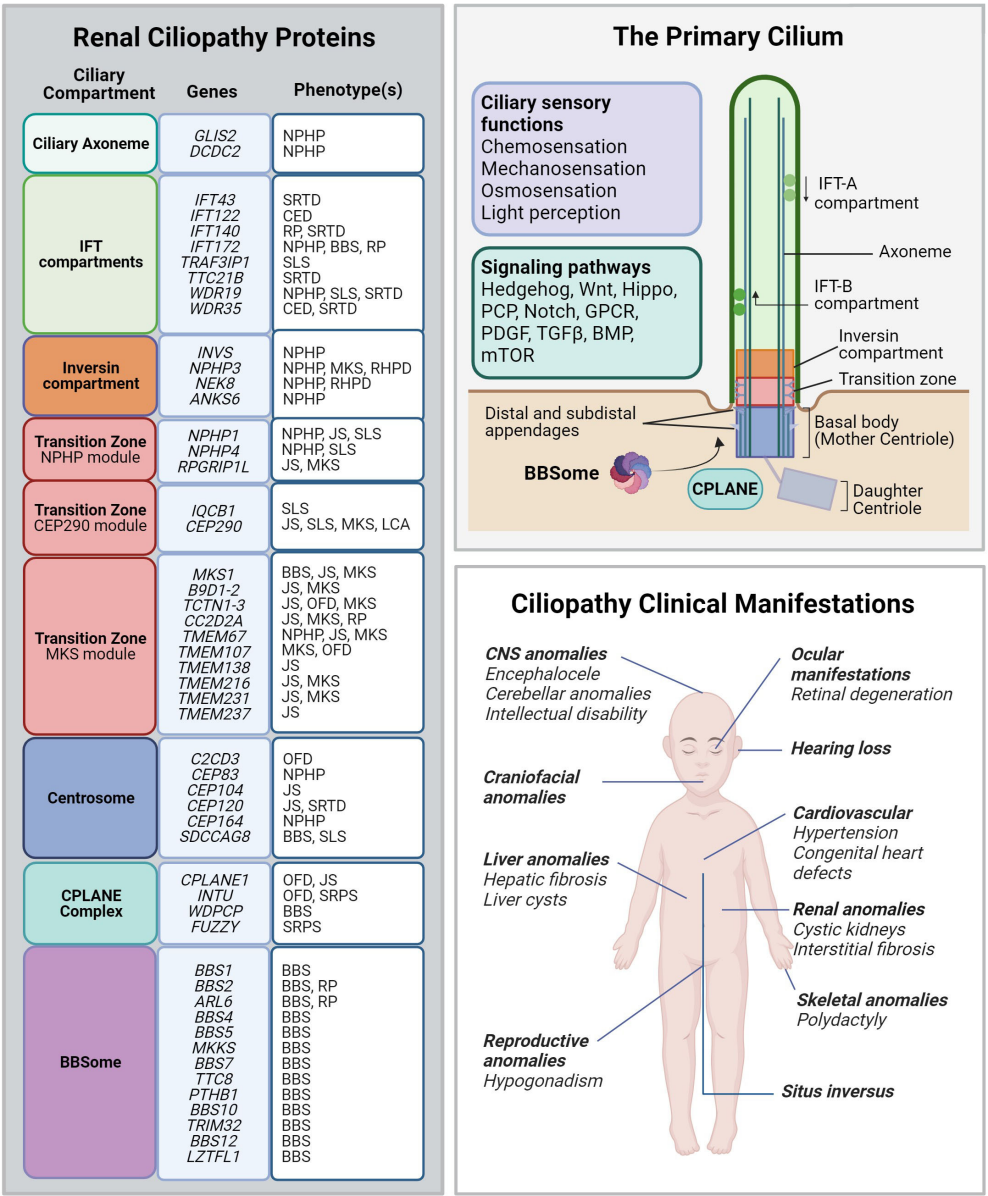
Renal ciliopathy gene-disease associations. *Left panel:* Genetic and phenotype information for renal ciliopathy gene-disease associations obtained from OMIM. Renal ciliopathies demonstrate tremendous genetic and phenotypic heterogeneity: pathogenic variants in many different genes can result in the same clinical ciliopathy phenotype, and different variants within the same gene can manifest in different phenotypes. The affected proteins in the same disease are often found to localize to the same ciliary compartment or converge on the same ciliary pathway. BBS, Bardet-Biedl syndrome; CED, Cranioectodermal dysplasia; JS, Joubert syndrome; LCA, Leber congenital amaurosis; NPHP, Nephronophthisis; OFD, Orofaciodigital syndrome; RHPD, Renal-hepatic-pancreatic dysplasia; RP, Retinitis pigmentosa; SLS, Senior-Løken syndrome. SRTD, Short-rib thoracic dysplasia. *Upper right panel:* Schematic depiction of the primary cilium and its protein compartments. The primary cilium regulates developmental programs from many sensory inputs (listed in the violet box) through multiple signaling pathways (listed in the green box). GPCR, G protein-coupled receptors; PCP, Planar cell polarity; PDGF, Platelet-derived growth factor; TGF-β, Transforming growth factor beta; BMP, Bone morphogenic protein; mTOR, Mammalian target of rapamycin. *Lower right panel:* Spectrum of the most common ciliopathic phenotypes discussed in the review.

The first indication that abnormalities of primary cilia can cause cystic kidney phenotypes, resembling human polycystic kidney disease (PKD), came from the studies of the Oak Ridge Polycystic Kidney (*orpk*) mouse (122, 123). Genetic analysis revealed that the *orpk* mouse harbored a hypomorphic allele in the *Ift88* gene, which is a component of the anterograde IFT-B complex (122, 124). The residual IFT88 activity from the *orpk* allele was sufficient for the generation of shorter, functionally impaired, primary cilia causing dilatation of proximal tubules, glomerular cysts as well as polydactyly and hydrocephalus (124–126). The studies of the *orpk* mouse have established the key role of the primary cilium in cystic kidney disease and started a new chapter in the exploration of the cilia role in human disease. Since then, hundreds of genes involved in the generation and function of the primary cilium have been identified. While the quest for mapping all genes relevant to human health is far from complete, the emerging picture revealed different functional protein compartments that are essential for the cilia role in mammalian development (**Figure 3**). In this ongoing process, new evidence has emerged, linking several key ciliary proteins to the regulation of the actin cytoskeleton. Below, we describe what is currently known about the role of actin regulation in different renal ciliopathies. Exploring the link between actin regulation and ciliopathies may change our present understanding of disease mechanisms and foster the development of novel treatments.

### Polycystic kidney disease

Autosomal-dominant polycystic kidney disease (ADPKD) is the most common cause of inherited kidney disease, with a reported incidence of 1:400 to 1:1,000 (127, 128). It primarily presents with renal cysts, liver cysts, and intracranial aneurysms (127). End-stage renal disease (ESRD) occurs in approximately 50% of patients by the age of 60 years. ADPKD is caused by heterozygous pathogenic variants in the genes *PKD1* (ADPKD1, OMIM #173900) and *PKD2* (ADPKD2, OMIM #613095), which comprise about 80% and 5-10% of total cases, respectively (127, 129, 130) (128, 131). The other cases of ADPKD either remain unsolved or are due to other genes [reviewed in (132, 133)].

Autosomal-recessive Polycystic Kidney Disease (ARPKD) occurs less frequently with a prevalence of 1 in 20,000 and is caused by biallelic variants in the *PKHD1* gene (ARPKD, OMIM #263200) (132). It is characterized by enlarged, echogenic kidneys with fusiform dilatation of the collecting ducts. Most patients progress to ESRD, however, there is some clinical variability. Notably, patients frequently have liver disease with dilated biliary ducts, congenital hepatic fibrosis and portal hypertension. The most characteristic ARPKD presentation is described in neonates, consisting of a history of oligohydramnios, massively enlarged kidneys, and the characteristic “Potter Sequence” with pulmonary hypoplasia that leads to respiratory insufficiency and perinatal death in 30% of affected newborns (134).

#### PKD molecular mechanisms of disease

*PKD1* and *PKD2* encode the transmembrane proteins Polycystin1 (PC1) and Polycystin2 (PC2), respectively. PC1 and PC2 form a heteromeric complex at the primary cilium, cell-cell junctions and cell-extracellular matrix interface (127, 129, 130). *PKHD1* encodes fibrocystin/polyductin, a ciliary membrane protein that is associated with the PC1/PC2 complex and localized to the primary cilium and basal body in renal tubular epithelial cells and bile duct cells (132, 135–137). The polycystin proteins are understood to function at the primary cilium and are important for maintaining the integrity of the renal epithelium. However, their precise cellular roles and the pathogenic mechanisms underlying cyst formation remain elusive (138). The polycystins are involved in mediating numerous cellular signaling pathways, including intracellular Ca^2+^, cAMP, mammalian target of rapamycin (mTOR), and others (127, 138). Initially, it was thought that polycystin complex at the primary cilium responded to external stimuli (e.g., fluid flow) to induce intracellular Ca^2+^ signaling (139), however, this role has been lately disputed (140). Moreover, its putative mechanosensory role at the primary cilium has also been contended (140). Others have suggested that ciliary-mediated proliferation pathways integral to cell division may be activated following loss of PC function, leading to unrestricted proliferation of renal tubular epithelial cells. The cystic growth can then produce a cascade of consequences including tubular obstruction, inflammation, ischemia, and metabolic disruption of renal tissue (127). On the cellular level, cystogenesis has been characterized by disruptions to apical-basal polarity, excessive WNT signaling or dysregulated planar cell polarity, aberrant extracellular matrix formation, and cell cycle regulation (141–143).

In ADPKD, it is hypothesized that a “second-hit” in the polycystin genes that reduces the PC function is required for cystogenesis in renal epithelial cells (127, 138, 144). Extensive research has shown that haploinsufficiency or complete loss of the polycystin protein function leads to a spectrum of cystic kidney phenotypes in a dosage-dependent manner (144, 145). Importantly, *in vivo* studies in mice show that ablation of the primary cilium in *Pkd1^-/-^* and *Pkd2^-/-^* mice suppresses cystogenesis and ameliorates the cystic phenotypes, suggesting that the polycystins may restrict cilium-originating signals required for cystogenesis (145). Although ciliary morphology is not grossly perturbed in PKD, as seen in other ciliopathy disorders, coordination of ciliary turnover and cell-cycle control may also be disrupted (146).

#### PKD proteins and actin regulation

Actin dynamics appear to play a role in PKD pathogenesis, as cystic cells are characterized by significant actin cytoskeletal disorganization and an accumulation of active RhoA at the basal body (112). Streets et al. showed that PC1 interacts with GTPase activating protein p190A RHOGAP, encoded by the *ARHGAP35* gene. Biallelic loss of *PKD1* results in the loss of p190A RHOGAP at the basal body and excessive centrosomal RhoA activity, implicating RhoA/ROCK signaling at the basal body in ADPKD pathogenesis (112). Cai and colleagues elucidated the essential role of the RhoA-YAP-c-Myc signaling axis in PKD cystogenesis (147). In *Pkd1* mutant mouse tissues, aberrant activation of RhoA triggers translocation of the transcription factor YAP to the nucleus. This results in the upregulation of c-myc promoting cell proliferation and renal tubule dilatation *in vivo*. Crucially, these effects were reversed with the inhibition of RhoA signaling (147). However, more recently, the involvement of transcriptionally-active YAP in ADPKD pathogenesis was disputed (148).

### NPHP and NPHP-related ciliopathies

Nephronophthisis (NPHP) is an autosomal-recessive renal ciliopathy that was initially described by Fanconi et al. in 1951 based on the disappearance of nephrons in the kidneys of affected children (149). Patients typically present with polydipsia, polyuria, microalbuminuria, and chronic tubulointerstitial nephritis; most patients progress to ESRD before the age of 30. Thus, NPHP is considered one of the most common hereditary causes of renal failure in the pediatric population and young adults (150). However, incidence estimates vary widely from 1 in 50,000 in Canada to 1 in 900,000 in the United States (151). In comparison to PKD, the kidneys are typically not enlarged and may be smaller in size. This explains the origin of the disease name, which derives from Greek and means “vanishing of the kidney”. Histological examination reveals a characteristic triad: loss of corticomedullary differentiation with occasional corticomedullary cysts, tubular atrophy with a thickening of the basement membrane, and interstitial fibrosis (150). Although cysts are found in about 70% of NPHP cases, they are smaller than in ADPKD and their presence is not necessary for an NPHP diagnosis (152). The first discovered and most common is nephronophthisis type 1 (NPHP1) (OMIM #256100), caused by biallelic variants in the *NPHP1* gene (often due to a homozygous deletion of the entire gene). NPHP1 accounts for 20-50% of all genetically solved NPHP cases (153–155). About one-third of NPHP1 cases are syndromic, often presenting with Senior-Løken syndrome, SLS (retinal degeneration) and Joubert syndrome, JS (cerebellar and oculomotor anomalies) (156). Currently, more than 25 genes have been identified as genetic causes of NPHP (155, 157). However, the majority (70%) of NPHP cases remain unsolved (157).

#### NPHP molecular mechanisms of disease

Many NPHP proteins localize to specific compartments at the primary cilium or centrosome and are involved in ciliogenesis or ciliary functions (**Figure 3**). Some NPHP proteins are directly involved in forming the basal body as well as facilitating its migration and docking to the cell membrane (158). Interestingly, many NPHP proteins involved in ciliopathies with either kidney or eye involvement cluster within the transition zone modules (156). The inappropriate accumulation of non-ciliary proteins in the cilium *proper* due to aberrant ciliary gating is hypothesized to contribute to disrupted ciliary function in NPHP and other renal ciliopathies (159). Many ciliary-mediated signaling pathways, including Hippo, cAMP, mTOR pathway and others, are implicated in NPHP disease pathogenesis [reviewed in (154)]. NPHP phenotypes are further associated with upregulation of pro-inflammation and pro-fibrotic pathways culminating in renal interstitial fibrosis and inflammation (150, 160, 161). The complex interactions between NPHP proteins and their roles in supporting ciliogenesis and ciliary function play a role in the phenotypic heterogeneity observed in NPHP-related ciliopathies.

#### NPHP proteins and actin regulation

Nephrocystins interact with actin and microtubule structures to coordinate ciliary signaling in renal epithelial cells, which mediates cell adhesion and cell division of renal tubular cells (162–164). Nephrocystin 1 (NPHP1) localizes to the basal body of the primary cilium and the apical surface of renal epithelial cells (160). NPHP1 functionally interacts with nephrocystin 4 (NPHP4) and nephrocystin 8 (NPHP8), forming the NPHP1-4-8 module within the ciliary transition zone in both polarized renal epithelial cells and at the centrosome of dividing cells. The NPHP1-4-8 protein complex is involved in cortical actin cytoskeletal organization and regulation of cellular apicobasal polarity during tubule morphogenesis and tissue maintenance (48, 161, 164). NPHP1 also interacts with proteins at focal adhesion complexes (164), which, in addition to enabling cell-extracellular matrix contacts, facilitate anchoring the basal body to the actin cytoskeleton in multiciliated cells (165). Loss of either NPHP1 or NPHP4 *in vitro* results in disrupted tight junction formation, aberrant ciliary function and loss of normal apicobasal polarity (166). In multiciliated *Xenopus laevis* cells, NPHP4 is involved in subcortical actin organization through interactions with formin DAAM1 and the PCP effector, Inturned (167).

Pathogenic variants in *INVS* result in an infantile form of NPHP (OMIM #602088), featuring large cystic kidneys and *situs inversus* in some patients. *INVS* encodes inversin, which forms the “inversin compartment” and controls switching of the canonical and PCP signaling pathways, believed to be important in renal development and tissue maintenance (168). Inversin is involved in regulating the cortical actin cytoskeletal network during mitosis, and loss of its function leads to mitotic spindle misalignment, defective PCP signaling, disrupted mitotic cell rounding, and loss of epithelial organization and integrity (169). Together, this gives rise to defective tubulogenesis as the cells fail to preserve oriented cell divisions and incorporate longitudinally along the developing nephron (170).

Aberrant RhoA activity at the centrosome appears to play a significant role in NPHP pathogenesis through activation of its downstream target, ROCK, and by promoting abnormal actin formation and activation of the Hippo pathway (95). The human kidney proximal tubular epithelial cells with an *NPHP1* knockdown and the renal tissue from *Nphp1* knockout mice exhibit increased levels of GEF-H1, an important RhoA GTPase effector factor in mammalian renal epithelium that mediates RhoA activation (171). The NPHP phenotype could be rescued in these mice by a knockdown of GEF-H1, which acts upstream of RhoA, thus reducing the RhoA activity and preventing renal cystogenesis, interstitial fibrosis and inflammation (171).

### Bardet-Biedl syndrome

Bardet-Biedl Syndrome (BBS) is a group of autosomal-recessive ciliopathies manifesting during early childhood. The diagnosis is based on the presence of several major diagnostic criteria (retinal dystrophy, obesity, intellectual disability, polydactyly, genital anomalies and renal malformations) but may also include other variable features affecting multiple organ systems (172). The prevalence ranges from 1 in 140,000 to 1 in 160,000 but has been reported more commonly on the island of Newfoundland, affecting 1 in 17,000 (173). Renal manifestations of BBS more closely resemble the fibrocystic phenotypes observed in NPHP, JS, and MKS, compared to PKD, and include dysplastic kidneys or nephronophthisis, occurring in about 40% of cases, and often leading to end-stage renal disease (174, 175). Renal disease in BBS is highly variable in presentation due to high genetic locus and allelic heterogeneity; however, the molecular basis behind this phenotypic variability remains poorly understood (175, 176). At least 26 different known genes have been identified as causes of BBS and likely contribute to the great variability in severity and clinical pleiotropy (177). Many of the BBS-causing genes encode the core BBS proteins forming the BBSome, a protein complex that facilitates ciliary vesicle trafficking toward and within primary cilium (**Figure 3**) (68). Disruption to any of the BBSome subunits can result in the clinical manifestations of BBS, providing strong evidence for the functional interdependency among all BBSome components.

#### BBS molecular mechanisms of disease

Defects to the BBSome can significantly alter the composition of the ciliary membrane leading to the loss, mis-localization or abnormal retention of membrane receptors and other molecules within cilium. For example, the photosensory protein rhodopsin is lost in the outer photoreceptor segment of *Bbs2, Bbs4* and *Bbs8* loss-of-function mutant mice, while other proteins inappropriately accumulate in the outer segment, leading to increased photoreceptor apoptosis and progressive retinal degeneration (178–180). Studies of the cells isolated from patients with BBS and pathogenic variants in *BBS1*, *BBS5* and *BBS10* demonstrate shorter cilia and abnormal SHH signaling; the latter likely arises due to inappropriate trapping of the key SHH component, Smoothened, within ciliary shaft (181). Downregulation of SHH signaling during development is linked to renal malformations and may contribute to cystogenesis in BBS and other renal ciliopathies (182). Suppression of *Bbs* transcripts in zebrafish leads to stabilization of β-catenin and inappropriate activation of canonical WNT pathway, that might contribute to the cystic renal phenotype in BBS (183). The BBSome is crucial for the localizations of G-protein-coupled receptors (GPCRs) at the primary cilium of certain neurons, and some of the phenotypic manifestations of BBS are thought to result from misplacement of specific GPCRs (71). Thus, like other syndromic ciliopathies, the pathogenic mechanisms of BBS are largely linked to defects in signaling pathways mediated by the primary cilium.

#### BBS proteins and actin regulation

Studies of *Bbs* mutant cells established a potential role of dysregulated actin polymerization in the pathogenesis of BBS (184). Defects in actin cytoskeletal regulation during development are implicated in renal malformations and renal cystogenesis in BBS. Renal medullary cells isolated from *Bbs4* and *Bbs6* deficient mice displayed a reduced percent of ciliated cells, shorter cilia, and abnormal actin fiber accumulation at the cell apex. Additionally, paucity of lamellipodia and filopodia was detected as well as loss of peripheral focal adhesions, the defects usually associated with decreased cell motility that was seen in the *Bbs* mutant cells. Inappropriate actin aggregation was attributed to significantly increased RhoA activity and dynamics of actin polymerization since treatment of mutant cells with selective RhoA kinase inhibitor Y27632 or actin polymerization inhibitor CytoD rescued actin aggregation phenotype. Importantly, these inhibitors also rescued ciliary length and increased percent of ciliated cells, pointing to the strong link between abnormal actin polymerization, increased RhoA activity and ciliary defects. Intriguingly, Bbs4 and Bbs6 proteins previously detected only at the basal body and in the cilium, also co-localized with the focal adhesions and governed their assembly. Thus, the results of Hernandez-Hernandez et al. support the hypothesis that BBS proteins regulate actin cytoskeletal arrangement through RhoA and focal adhesion dynamics, functions that are now recognized as integral to ciliogenesis (184).

### Meckel-Gruber syndrome

Meckel-Gruber syndrome (MKS, OMIM #24900) is a perinatally lethal autosomal-recessive ciliopathy that presents with a constellation of severe developmental anomalies including neural tube defects, skeletal malformations, congenital heart defects, liver anomalies, and enlarged cystic dysplastic kidneys (152, 185). The worldwide incidence for MKS is 1 in 13,250 to 1 in 140,000 live births (186). It is the ciliopathy with the most severe presentation, often arising from biallelic full loss-of-function variants in ciliary genes that lead to total or significant loss of ciliary function (152). Several genes have been associated with MKS, including *MKS1*, *TMEM67*, *CEP290*, *CC2D2A*, and *TMEM216*, many of which encode proteins that localize to the transition zone and are essential for basal body docking (152, 187). Most MKS-causing genes are also associated with milder ciliopathy phenotypes, causing NPHP or JS, suggesting that these disease entities exist along an “allelic spectrum” and depend on the nature of the underlying genomic variant (188).

#### MKS molecular mechanisms of disease

Many of the MKS proteins converge on the same pathway during ciliogenesis, and their loss-of-function models have demonstrated a critical role in mediating basal body anchoring in association with actin cytoskeletal dynamics (187, 189–191). The transition zone protein TMEM67 (also known as Meckelin), encoded by *TMEM67*, plays a crucial role in ciliogenesis. Biallelic truncating pathogenic variants in *TMEM67 (MKS3)* commonly lead to Meckel syndrome (MKS3, OMIM #607361); however, “milder” *TMEM67* allelic combinations (e.g., biallelic missense variants) lead to less severe phenotypic presentations such as JS or isolated NPHP (192, 193). TMEM67 and MKS1 interact together and are required to mediate basal body docking to the apical cell surface (189, 194).

#### MKS proteins and actin regulation

The involvement of MKS proteins in actin regulation is only beginning to emerge. The TMEM67 is suggested to promote actin-cytoskeletal reorganization required for basal body docking by regulating RhoA-ROCK signaling (190). Loss of TMEM67 or MKS1 leads to disrupted ciliogenesis and impaired epithelial morphogenesis (189). Similarly, loss of *TMEM216* in human fibroblasts or its knockdown in zebrafish was shown to cause hyperactivation of RhoA, hypothesized to disrupt the required actin reorganization at the cell apex preventing normal basal body docking (187).

### Skeletal ciliopathies with renal involvement

Approximately 10% of syndromic NPHP cases present with skeletal anomalies often found alongside liver, eye, and CNS anomalies (156). Short-rib thoracic dysplasia with and without polydactyly (SRTD) are a group of skeletal ciliopathy disorders with shared features of polydactyly, shortening of the long bones and severe rib and thoracic malformations (152, 195). The most common forms of SRTD are Jeune asphyxiating thoracic dysplasia (JATD), Ellis–van Creveld syndrome, Sensenbrenner syndrome, Mainzer-Saldino syndrome (MZSDS), and short-rib polydactyly syndromes (SRPS) (196). Polycystic kidneys are occasionally detected in patients with Oral-facial-digital syndromes (OFD), which are primarily characterized by dysmorphic craniofacial and oral anomalies and polydactyly (197). Ciliopathies with skeletal malformations often arise due to defects in genes encoding for the IFT-A and IFT-B components or the dynein motor arms of the primary cilium, leading to defects in ciliary function, including mediating SHH signaling (**Figure 3**) (152). Defects to the IFT-A complex of proteins (e.g., *WDR35, IFT122, IFT43, IFT172, IFT140* and *WDR19*), can present with renal disease phenotypes such as chronic renal failure, NPHP, and renal cysts (63, 198–203) and, in some cases, isolated NPHP or NPHP-like nephropathy (63). Although the underlying mechanisms are not well characterized, several groups have posited that IFT proteins may be regulating planar cell polarity in the elongating renal tubules, although this supposition requires experimental confirmation. There are over 16 genes associated with OFD phenotypes, however, the X-linked OFD1 (OMIM # 300170) is the most common (~50% of all OFD cases) and causes polycystic kidney disease in half of affected patients (152, 204, 205). OFD1 protein localizes to the centrosome and is essential for recruiting the distal appendage proteins during ciliogenesis (206). Recent research uncovered the expanded role of OFD1 in microtubule organization and cell-cycle regulation as well actin filament branching (207, 208).

#### Skeletal ciliopathies caused by distortion of CPLANE function

A special subset of skeletal ciliopathies featuring renal malformations is caused by defects in the Ciliopathy and Planar Cell Polarity, *CPLANE*, genes (209). Originally discovered in *Drosophila* (210), where these genes are known as “Planar cell polarity effectors” and participate in PCP signaling, vertebrate homologs of *inturned*, *fuzzy* and *fritz* (*WDPCP*) regulate ciliogenesis. Their depletion in frogs led to significantly shorter cilia (211); homozygous mutations of *Fuzzy*, *Inturned* and *Wdpcp* in mice caused stunted sparse primary cilia and features of classical ciliopathies such as craniofacial defects, polydactyly, and dysplastic kidneys (212–214). CPLANE proteins interact with each other (215), localize to the basal body and control important aspects of vesicular trafficking to the basal body (209, 216). Comprehensive tandem affinity purification studies revealed interactions with various ciliary proteins including IFT43 and WDR35 (209) [both cause Sensenbrenner syndrome (201, 217)]. They also interact with and form a functional complex with two additional proteins, Joubert syndrome 17 encoded by the *JBTS17* gene (also known as CPLANE1) (209) and a small GTPase effector RSG1 (209). Pathogenic variants in *CPLANE1* cause JS and OFD, often presenting with renal involvement (218). Variants in *FUZ, INTU, and WDPCP* are associated with embryonically lethal cases of SRPS, OFD syndrome, and neural tube defects (209, 219, 220); renal hypoplasia was described in some of these patients.

#### CPLANE and actin regulation

**Figure 4:**
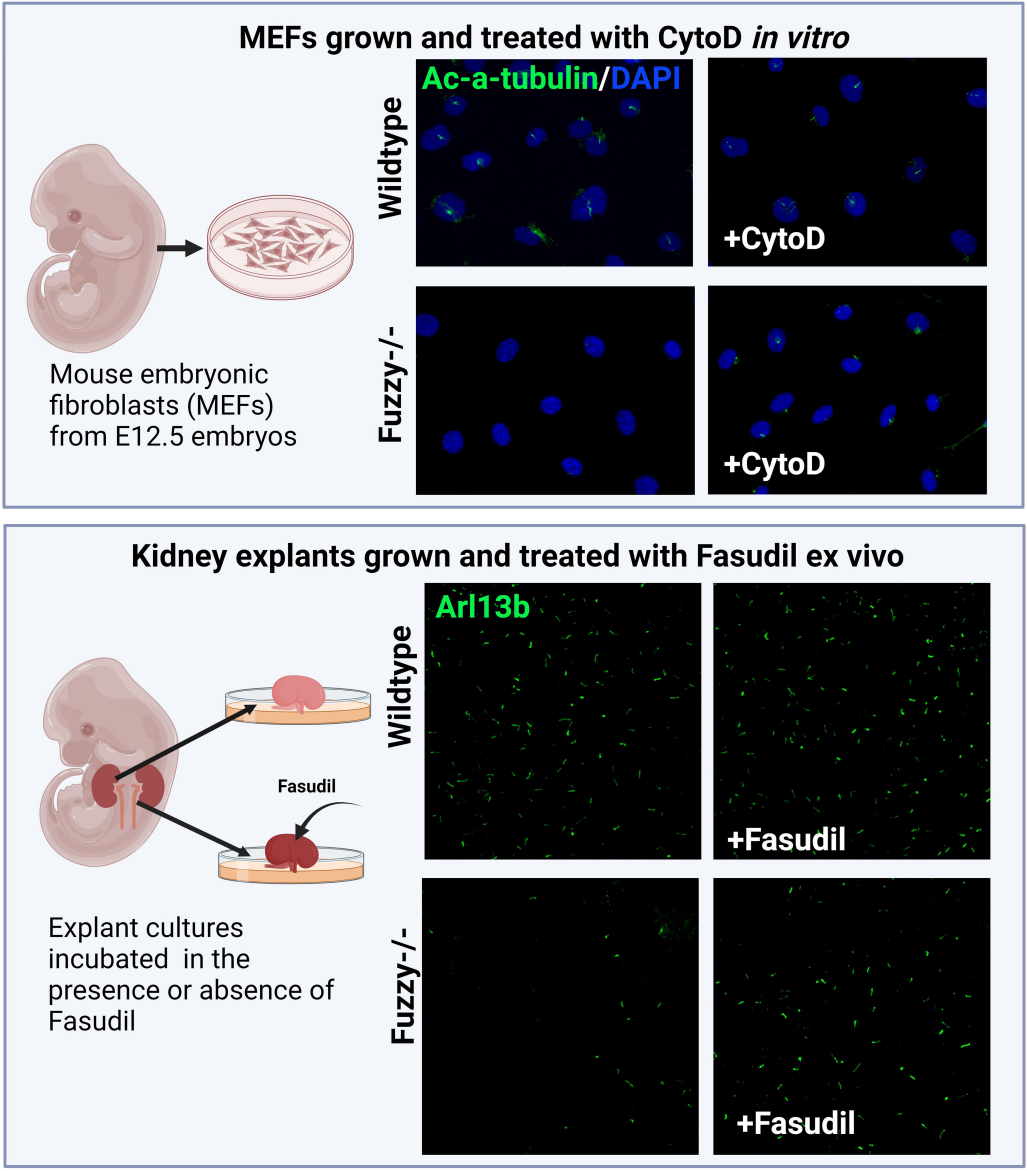
Inhibition of actin signaling pathway and/or polymerization rescues ciliogenesis in a CPLANE ciliopathy model. *Upper panels:* Schemata of generating mouse embryonic fibroblasts (MEFs) from E12.5 embryos. Wildtype MEFs are ciliated at ~ 80-85% after 24 hours of starvation *in vitro* whereas *Fuzzy-/-* MEFs lack cilia. Treatment of unciliated *Fuzzy-/-* MEFs with 0.5µM of CytoD leads to 60% ciliated mutant cells and no effect on the wildtype cells; cilia were visualized with anti-acetylated-α-tubulin antibody and 4′,6-diamidino-2-phenylindole to stain nuclei (unpublished data). *Lower panels*: Schematic depiction of an experiment in which embryonic kidney explants from E12.5 wildtype and *Fuzzy-/-* embryos were cultured *ex vivo* for 72 hours in the presence or absence of 2.5µM Fasudil followed by a whole mount tissue immunostaining with anti-Arl13b antibody to detect primary cilia. Treatment with Fasudil elongates ciliary length in mutant tissues with no effect on the wild-type tissues (unpublished data).

Knockdown of *Fuzzy* or *Inturned* reduces the thickness of the actin cortical network in multiciliated *Xenopus* cells (211), implying that in this context, CPLANE proteins might function as positive regulators of actin polymerization. Similarly, mouse *Wdpcp* mutant cells display thinner actin-based stress fibers and minimal actin-based membrane protrusions; the latter affects cell motility (213). The effect of WDPCP on actin was traced to its interaction with actin regulatory protein Septin 2 (213). However, Drosophila CPLANE homologs function to restrict actin polymerization in the wing cells and their loss leads to an excessive actin cytoskeleton consistent with the negative actin regulatory function in the fly cells. In agreement with this, we recently observed both excessive RhoA activity and increased actin polymerization at the basal body of *Fuzzy* mutant cells (unpublished data). Importantly, ciliogenesis could be rescued with CytoD or ROCK inhibitors *in vitro* and *ex vivo* (**Figure 4**). Thus, the accumulated evidence suggests that CPLANE proteins affect ciliogenesis via differential effects on actin polymerization in various cells, and this may contribute to the ciliopathic phenotypes of skeletal/renal ciliopathies.

### Actin-involvement in ciliopathy-associated diseases

#### Townes-Brock syndrome 1

The majority of the pathogenic mechanisms of renal ciliopathies described above are associated with increased centrosomal actin, however, Townes-Brock syndrome 1 (TBS1, OMIM #107480) features reduced actin polymerization. TBS1, an autosomal-dominant disorder, results from heterozygous loss-of-function variants in the *SALL1* gene and features ciliopathy-spectrum phenotype with renal abnormalities often leading to ESRD (221). *SALL1* encodes a transcription factor that interacts with CEP97 and CP110, two negative regulators of ciliogenesis, and removes them from the mother centriole, thereby favoring ciliogenesis. Loss of SALL1 function leads to defects in ciliogenesis and SHH signaling in patient-derived fibroblasts (221). Recently, Bosal-Basterra et al. reported that truncated SALL1 protein interacts with LUZP1, a leucine-zipper motif-containing protein that localizes around centrioles and binds to filamentous actin (222). Loss of *LUZP1* reduces F-actin and increases ciliogenesis. Importantly, interactions between mutant SALL1 and LUZP1 target LUZP1 for degradation leading to the disruption of both ciliogenesis and SHH signaling through reduced F-actin polymerization, indicating that dysregulation of centrosomal actin cytoskeleton contributes to the pathogenesis of TBS (222).

#### Lowe syndrome

Lowe syndrome (OMIM # 309000) is an extremely rare and severe X-linked oculo-cerebro-renal disorder that results from pathogenic variants in the *OCRL* gene encoding inositol polyphosphate 5-phosphatase OCRL-1. The proximal tubular dysfunction leads to renal failure in the second or third decade (223). Some patients with *OCRL* variants present with Dent disease type 2, which is thought to be a milder presentation of classical Lowe syndrome (223). OCRL-1 is involved in membrane and endosomal trafficking. It also binds Rho GTPases to modify F-actin polymerization important for phagocytosis, cell migration, cell polarity, and ciliogenesis (223–225). In *OCRL-1* mutant cells, actin polymerization is decreased and primary cilium formation and function are disrupted (226) due to defects in ciliary vesicle processing during ciliogenesis (225, 227).

Overall, the studies of renal ciliopathies described above indicate that many renal ciliopathy proteins localize to the basal body and participate in the complex interactions that organize centrosomal actin remodeling required for the successful formation of primary cilium. These emerging data have uncovered a frequent distortion of actin regulation and remodeling in pathogenesis of renal ciliopathies.

## Primary cilium in renal cell carcinomas

Renal cell carcinomas (RCC) frequently present with cystic kidneys, prompting studies of primary cilia in the context of RCC (228, 229). Germline heterozygous pathogenic variants in the Von Hippel-Lindau (*VHL*) gene is the most common genetic cause of RCC, where renal cysts often precede tumorigenesis (230). *VHL* encodes a tumor suppressor protein VHL that localizes to the Par3-Par6-aPKC complex at the primary cilium to facilitate ciliogenesis by stabilizing and orienting microtubule formation (228, 231, 232). VHL-deficient cells are not ciliated and exhibit defects in cellular polarity and cell-cycle regulation, leading to uncontrolled cell proliferation (233, 234). Interestingly, VHL also acts as a E3-ubiquitin ligase, and in this capacity, targets the ciliary disassembly factor Aurora Kinase A (AURKA) for degradation (235). Moreover, loss of VHL function results in high levels of AURKA, affecting stability of the microtubular axoneme and leading to disassembly of the primary cilium followed by development of cystic kidneys and RCC (235). Other ciliary genes involved in RCC have been recently reviewed elsewhere (236). Tuberous sclerosis TSC is an autosomal-dominant multi-system disorder that results from pathogenic variants in the tumor suppressor genes, *TSC1* and *TSC2*, leading to aberrant activation of the mTOR pathway (237) and often presenting with renal cysts associated with cilia disruption (238). Currently, the role of actin regulation at the primary cilium assembly and disassembly in renal cancers and associated cystic phenotypes have not been studied.

## Potential therapies

Renal ciliopathies are a significant cause of chronic kidney disease and ESRD. Currently, the only long-term treatments for kidney manifestations are renal replacement therapy through hemodialysis and kidney transplantation. High frequency of ADPKD, that affects more than 12 million people worldwide, prioritized significant scientific and financial resources to develop various treatment options. However, despite a significant effort, Tolvaptan has so far been the only FDA-approved medication for ADPKD (239). On the other hand, there are currently no approved treatments for other renal ciliopathies. Recent clinical trials for BBS were limited to obesity and hyperphagia treatment by Setmelanotide (a selective melanocortin-4 receptor agonist) with no focus on treating kidney phenotypes (240).

Drugs that prevent cilia disassembly via axoneme stabilization through histone deacetylase inhibition have also been tested in several pre-clinical models (241–243). Histone deacetylase 6 (HDAC6) inhibitors such as ACY-1215, Tubacin, Trichostatin A (TSA), Valproic acid (VPA) and Nicotinamide have shown to ameliorate cystic kidney disease in *in vitro* and *in vivo* models, however, some of these drugs have uncertain safety profiles and are yet to be tested in clinical trials. Excessive cell proliferation in the VHL-dependent RCC cells was rescued by treatment with alisertib, an AURKA−specific chemical inhibitor (244), however, ciliary integrity in the treated RCC cells was not investigated. Other treatments that target cellular metabolism (e.g., basal body localized-AMPK agonists such as Metformin), inflammation, EGF signaling, mTOR inhibitors, HDAC inhibitors, CDK inhibitors, Hedgehog agonists, somatostatin analogues, in addition to gene therapy via microRNAs, are all extensively reviewed elsewhere (243).

Recent evidence on the role of the RhoA/ROCK pathway in ciliogenesis heightened interest in the studies of actin regulation in relation to ciliopathies. Actin polymerization inhibitors such as Cytochalasin D or selective ROCK inhibitors Y27632 have shown to rescue cilia defects to some degree in different ciliopathy disease models (39, 101, 112, 184) (**Figure 4**). Importantly, inhibition of RhoA signaling with Y27632 suppresses cystogenesis in both the 3D culture of *Pkd1* mutant collecting duct cells and in *Pkd1* mutant mouse kidneys *in vivo* (147). However, their use in humans is difficult to envision due to their toxicity. On the other hand, ROCK inhibitor Fasudil, that has a reduced toxicity and a more favorable safety profile, was clinically approved for use in humans as a systemic ROCK inhibitor to treat cerebral vasospasm in Japan and China (245). Although it is still not FDA-approved, Fasudil is being used in several clinical trials addressing cardiovascular disorders (246, 247), Parkinson’s disease, Amyotrophic Lateral Sclerosis, and other conditions (https://clinicaltrials.gov). Given the persistent deregulation of RhoA signaling and actin polymerization in many models of renal ciliopathies, including ADPKD, NPHP and BBS, it would be reasonable to test Fasudil in human trials to treat ciliopathies, e.g., ADPKD or NPHP. With the advent of new inhibitors of actin regulatory pathways that can be targeted to specific tissues and have acceptable safety profiles, these molecules can be harnessed for their potential use in treating ciliopathies postnatally.

## Conclusions

The primary cilium is an enigmatic organelle central to the development and homeostasis of all human organs. Dysfunction of primary cilium perturbs numerous cellular processes, negatively affecting tissue morphogenesis and tissue maintenance causing a constellation of severe, frequently congenital, phenotypes in humans, known as ciliopathies. Pathogenic variants in over 180 genes have been identified to date as the cause of human ciliopathies, which often present as malformations of the kidney. However, analysis of cohorts of patients with specific ciliopathies shows that only a fraction of genes has been identified. Assembly of clinically well-characterized patient cohorts and the use of a comprehensive genome-wide molecular diagnostic testing are critical if we are to identify the hidden genetic contributors and will pave the way towards improved diagnostic criteria and elucidation of the molecular mechanisms which will allow personalized therapies.

Analysis of the mechanisms causing ciliopathies has revealed an unexpected key role of actin cytoskeletal dynamics in ciliogenesis and ciliary function. Moreover, it is becoming clear that disruption to these mechanisms plays a part in the pathogenesis of various classes of renal ciliopathies, including ADPKD, NPHP, or BBS. However, whether actin dysregulation is a driving force in the cellular phenotype or merely is a consequence of an upstream mechanism that alters actin dynamics remains unknown. Importantly, deregulation of basal body actin network affects both cilial assembly and disassembly. For example, loss of cilia in renal cancer appears to be driven by the heightened activity of the cilium disassembly, but it remains unknown whether actin deregulation at the base of the cilium contributes to the cilia loss in RCC. Regardless of the precise nature of the cause-and-effect relationship, persistent actin network disorganization in the cells of patients affected with ciliopathies may offer an opportunity to identify novel preventative and restorative treatments.

The cellular and molecular mechanisms underlying renal ciliopathies are currently under intense investigation. The expanding complexity of the genetic errors and phenotypic consequences and the basis for tissue specificity associated with specific genes promise to be an exciting journey.

## Author contributions

RK: Conceptualization, Writing – original draft, Writing – review & editing. ZS: Conceptualization, Writing – original draft, Writing – review & editing. TK: Funding acquisition, Supervision, Writing – review & editing, Conceptualization. ET: Conceptualization, Funding acquisition, Resources, Supervision, Writing – original draft, Writing – review & editing.
